# Evaluation of efficacy and recurrence for anti-vascular endothelial growth factor therapy in idiopathic choroidal neovascularization

**DOI:** 10.1186/s12886-020-01390-4

**Published:** 2020-03-19

**Authors:** Qianru Wu, Xiaoyong Chen, Kang Feng, Yuling Liu, Chun Zhang, Lin Zhao

**Affiliations:** Department of Ophthalmology, Beijing Key Laboratory of Restoration of Damaged Ocular Nerve, Peking University Third Hospital, No. 49 North Garden Road, Haidian District, Beijing, 100191 People’s Republic of China

**Keywords:** Idiopathic choroidal neovascularization, Intravitreal injection, Anti-VEGF therapy, Prognosis, Recurrence

## Abstract

**Background:**

This study aimed to evaluate the visual and morphological outcomes of intravitreal anti-VEGF therapy and the recurrence for idiopathic choroidal neovascularization (ICNV).

**Methods:**

This retrospective study included 35 patients (35 eyes) with ICNV from July 2012 to October 2017. All patients received 1 intravitreal anti-VEGF injection followed by pro re nata injections until there was no sign of ICNV activity. This was defined as the first follow-up period. To evaluate ICNV recurrence, we continued to follow-up 27 of the 35 patients for at least 2 years after the initial diagnosis, and the longest follow-up period was 5 years. Additional injection was performed when ICNV recurred. Best corrected visual acuity (BCVA) and central retinal thickness (CRT) were recorded and morphological improvement in optical coherence tomography (OCT) was assessed. Parameters that affect prognosis and recurrence were analysed.

**Results:**

The mean follow-up period was 168.0 ± 34.82 weeks. Mean BCVA improved from 56.20 ± 14.13 letters at baseline to 73.31 ± 12.57 letters (*P*<0.01); Mean CRT decreased from 353.6 ± 98.70 μm at baseline to 273.1 ± 53.56 μm (*P* < 0.001) at the end of the first follow-up period. Better baseline BCVA indicated a better morphological improvement (*P* = 0.026) in OCT: the lesion had completely subsided with recovery of the foveal contour. Those with high baseline BCVA (more than 60 letters) showed significant resolution of CNV lesions (*P* = 0.036). ICNV recurred in six patients (22.2%), 1 of whom experienced 2 recurrences. The mean timing of recurrence was 90.83 ± 49.02 weeks after diagnosis. There was no significant correlation between ICNV recurrence and the morphological improvement (*P* = 0.633). The final BCVA in patients with recurrence did not differ from that in patients without recurrence (*P* = 0.065).

**Conclusions:**

Intravitreal anti-VEGF therapy on a pro re nata basis was effective for treating ICNV. High baseline BCVA indicated a better prognosis. Re-treatment with anti-VEGF could effectively lead to resolution of recurrent ICNV. Disease recurrence had no significant effect on final visual prognosis and had no correlation with the morphological improvement during treatment, suggesting that follow-up for subsequent monitoring should be performed in all ICNV patients.

## Background

Choroidal neovascularization (CNV) is a proliferative disease and a common cause of visual impairment. Originating in choroidal blood vessels, this disease is usually associated with other fundus pathology [1]. It has been suggested that when abnormal blood vessels originating from the choriocapillaris break through the Bruch membrane and grow in the retinal pigment epithelium (RPE) or infiltrates into the subretinal pigment epithelium or subretinal space [2, 3], they can result in CNV. CNV that develops in younger patients who are less than 50 years old is usually attributed to pathologic myopia, angioid streaks, trauma, cytomycosis, central serous chorioretinopathy (CSC) and other hereditary ocular diseases [4, 5]. However, the cause of CNV still remains unclear in a large number of younger patients. In this subset of the disease, no apparent primary ocular or systemic pathology can be detected, and such cases are defined as idiopathic choroidal neovascularization (ICNV) [6, 7].

Currently, there are several available treatment options for ICNV, including intravitreal injection of anti-vascular endothelial growth factor (VEGF) such as ranibizumab, aflibercept and the off-label use of bevacizumab, which has previously been shown to be effective in the treatment of ICNV [7–10]. Since the majority of ICNV patients are under the age of 50 [11], it is necessary to study the long-term efficiency of this treatment. The follow-up period in previous studies has been relatively short, usually within 2 years [7, 8, 10]. Therefore, longer follow-up periods were needed for ICNV patients to investigate the prognosis as well as the possibility of disease recurrence.

Optical coherence tomography (OCT) is widely used to evaluate the prognosis and monitor the recurrence of ICNV because of its minimum side effects [12]. Additionally, retinal morphological change from OCT, which represents the retinal fluid, is convenient for clinicians to monitor the progression of disease. However, few studies have taken morphological parameters of OCT into account when evaluating the prognosis of the disease. The aim of our study is to evaluate the efficacy of intravitreal anti-VEGF therapy and the incidence of CNV recurrence in ICNV patients during a relatively long follow-up period, with a focus on morphological changes in OCT.

## Methods

This retrospective study was performed at Peking University Third Hospital, Beijing, China. Thirty-five patients (35 eyes) with ICNV were enrolled from July 2012 to October 2017. All procedures conformed to the tenets of the Declaration of Helsinki and the study was approved by the Medical Science Research Ethics Committee. Informed consent was obtained. All patients were less than 50 years of age (range: 17–50). All subjects underwent a comprehensive pre-operative examination, which included best-corrected visual acuity (BCVA) using the Early Treatment Diabetic Retinopathy Study (ETDRS) chart, intraocular pressure measured by the Goldman method, slit-lamp biomicroscopy, colour fundus photography, spectral-domain OCT (SD-OCT; Spectralis-OCT, Heidelberg Engineering, Heidelberg, Germany), and fluorescein angiography (ff450, Carl Zeiss GmbH, Oberkochen, Germany). With SD-OCT images, the quantitative and morphologic measurements of the fovea were evaluated. Central retinal thickness (CRT) was recorded as the central 1-mm radius of the macular map, which represented the distance between the vitreoretinal interface and the retinal pigment epithelial-Bruch’s membrane complex [13], and the presence of macular oedema and intraretinal/subretinal fluid was evaluated. All patients were diagnosed with ICNV according to fluorescein angiography and optical coherence tomography, which showed CNV lesions in the foveal area and increased thickness of the retina. No other ocular diseases were found during the follow-up period. Patients who had CNV caused by pathological myopia (refractive index>6D), uveitis, angioid streaks, trauma, cytomycosis, central serous chorioretinopathy (CSC) or other hereditary ocular diseases were excluded. Patients who had received other treatments before the anti-VEGF injections, such as laser, glucocorticoid, and ocular surgery were also excluded from the study.

After one intravitreal ranibizumab (IVR, 1.25 mg/0.05 ml, Novartis, Schweiz) or bevacizumab (IVB, 1.25 mg/0.05 ml, Genentech, USA) injection, patients were assessed monthly and received pro re nata (PRN) treatments until there was no sign of CNV activity based on the following criteria: no presence of subretinal/intraretinal fluid, no persistent/recurrent retinal haemorrhage or no drop in visual acuity of 5 letters, or the lesion became compact and only cicatricial CNV remained compared to the previous visits on OCT [14, 15]. Either bevacizumab or ranibizumab was used as monotherapy. The follow-up visits were scheduled every four weeks and we defined this period as the first follow-up period. Subsequently, when there was no sign of CNV activity, the follow-up interval was extended to a maximum of 12 weeks to evaluate recurrence. Recurrence criteria included [9]: (i) significant drop in visual acuity; (ii) new macular haemorrhage; and (iii) recurrence of intraretinal/subretinal fluid of cystic maculopathy on OCT. Additional intravitreal anti-VEGF on a PRN basis was provided when recurrence was detected, and the treatment interval was reduced to four weeks until no signs of CNV activity could be observed. During the first follow-up period, the shortest follow-up time was 12 weeks and the longest was 83.9 weeks. Eight patients failed to complete the follow-up period and were excluded from our study when recurrence was investigated. The other patients were subsequently followed-up for at least 2 years. The longest follow-up period was 5 years.

Ophthalmological examinations were performed at each visit after intravitreal injections, including BCVA testing, intraocular pressure testing, fundus photography, and SD-OCT to evaluate the effects of the treatment. Data collected included age, gender, type of anti-VEGF therapy, number of injections, ICNV duration, baseline BCVA, baseline CRT and the resolution of BCVA and CRT at the end of first follow-up period. Efficacy of the anti-VEGF therapy were evaluated and patients were classified into two groups according to the type of drugs. Then, patients were classified into two groups according to the morphological changes based on OCT results after the first follow-up period: patients with morphological improvement and patients without morphological improvement. According to the OCT results, morphological improvement was defined as follows: compared to the baseline, there was complete resolution of intraretinal/subretinal fluid and macular oedema with recovery of foveal contour. Those who without morphological improvement were defined as follows: although intra/subretinal fluid had subsided and the lesion had become compact, there was still cicatricial CNV remaining and the foveal contour was still abnormal. Factors that affected the prognosis were analysed. After the first follow-up period, BCVA and OCT were performed every 12 weeks to observe the recurrence of the disease. Then, patients were divided into two groups on the basis of the ICNV recurrence: patients with recurrence and patients without recurrence. Various characteristics, including age, number of injections, baseline BCVA, baseline CRT, final BCVA and duration of the disease, were compared between these 2 groups to evaluate the predictors for recurrence and influence of recurrence on prognosis.

Statistical analysis was performed using SPSS 23.0 (SPSS Inc., USA). Data were expressed as the means ± standard deviation. Normality was tested using the Shapiro-Wilk test. Follow-up and baseline data were compared using the Wilcoxon Signed Ranks Test. Comparisons of age, gender, type of anti-VEGF therapy, number of injections, ICNV duration, baseline BCVA, baseline CRT, the resolution of BCVA and CRT, and BCVA after treatment between the two groups were performed using the Chi-square test for categorical variables, independent sample t-test or the Mann–Whitney U test. Univariate analysis was performed using a logistic regression model. A *P* value of < 0.05 was considered statistically significant.

## Results

Thirty-five eyes (35 patients; 14 males and 21 females) with ICNV were included in this study. The mean age was 35.94 ± 9.471 years old (range: 17–50). The duration of the first follow-up period was 24.25 ± 15.97 weeks (range: 12–83.9). The average number of injections was 2.40 ± 0.78 times (range: 1–4). Five patients (14.3%) had only 1 injection. Twelve patients (34.3%) had 2 injections and seventeen patients (48.6%) had 3 injections.

### Visual outcomes and changes in OCT

Table 1 shows the changes in patients after anti-VEGF treatment. Overall, the baseline BCVA improved from 56.20 ± 14.13 letters to 73.31 ± 12.57 letters (*P* < 0.001) at the end of the first follow-up period. The baseline CRT decreased from 353.6 ± 98.70 μm to 273.1 ± 53.56 μm (*P* < 0.001) at the end of the first follow-up period. As shown in Table 2, there was no significant difference between the IVB and IVR groups in BCVA (*P* = 0.529) or CRT (*P* = 0.270) at the end of the first follow-up period.

**Table 1:** Changes in visual acuity and OCT after intravitreal anti-VEGF treatments in patients

Times	Eyes	BCVA (letters)	CRT(μm)
Baseline	35	56.20 ± 14.13	353.6 ± 98.70
End of the first follow-up period	35	73.31 ± 12.57	273.1 ± 53.56
Z		−4.481	−4.574
*P*^*a*^		< 0.001	< 0.001

^a^ Statistical analysis performed by the Wilcoxon Signed Ranks Test

**Table 2:** Comparative Analysis of Bevacizumab and Ranibizumab groups

Agent	Eyes	Number of injections(times)	Baseline BCVA(letters)	Baseline CRT(μm)	BCVA at the end of the first follow-up period(letters)	CRT at the end of the first follow-up period (μm)
Bevacizumab(IVB)	10	2.50 ± 0.85	53.30 ± 16.69	317.5 ± 86.41	75.10 ± 11.72	274.6 ± 26.95
Ranibizumab(IVR)	25	2.36 ± 0.757	57.36 ± 13.17	368.0 ± 101.2	72.60 ± 13.05	272.4 ± 61.56
*P*(IVB vs IVR)		0.438^b^	0.451^a^	0.141^b^	0.529^b^	0.270^b^

^a^ Statistical analysis performed by the sample t-test

^b^ Statistical analysis performed by the Mann–Whitney U test

### Comparison between subgroups according to the morphological change

**Fig. 1:**
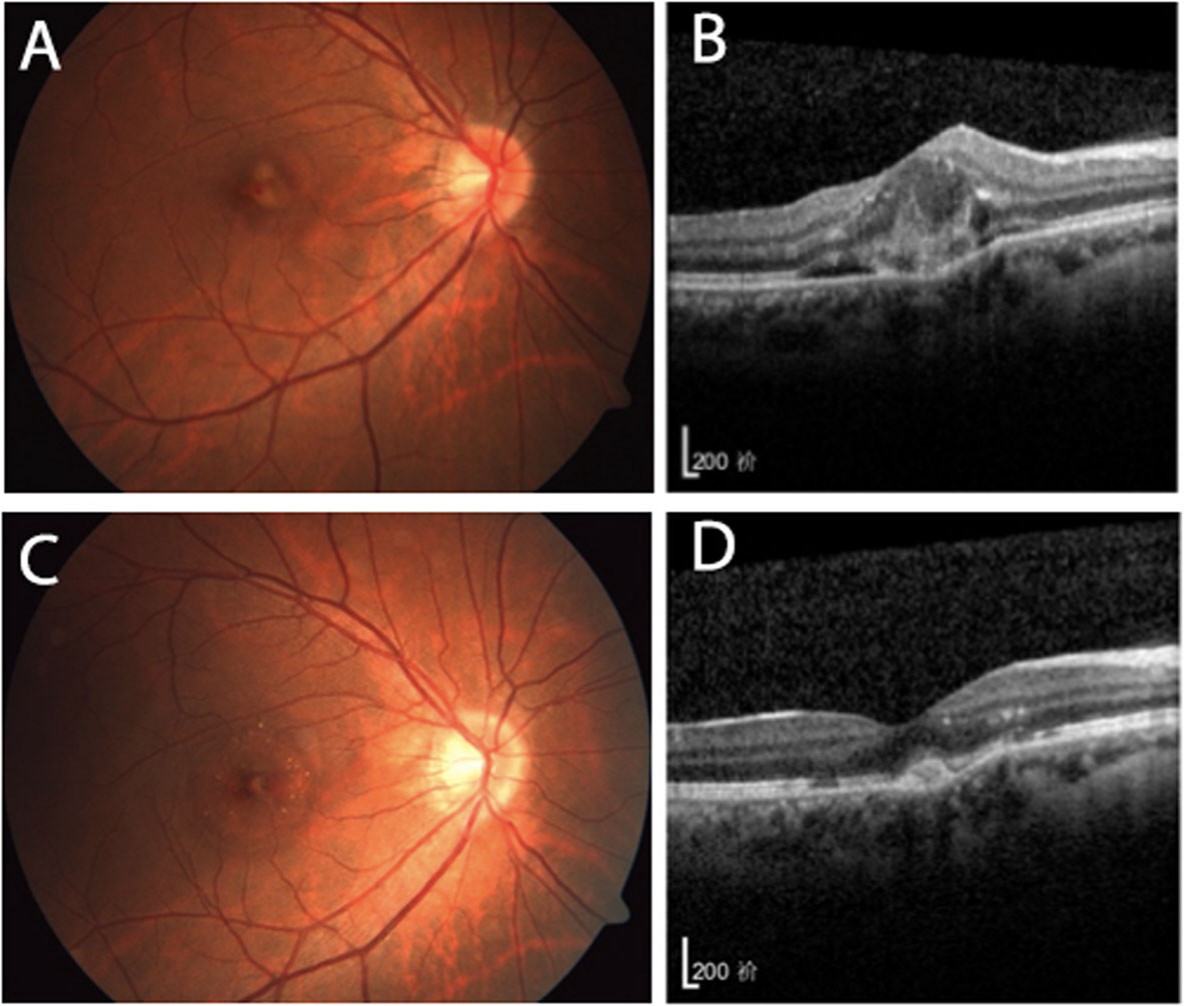
Left eye of a patient with morphological improvement after the first follow-up period. At baseline, BCVA was 54 letters and the colour fundus photo (**a**) showed a pigmentation disorder of the macular centre. SD-OCT (**b**) showed an ICNV lesion and intraretinal fluid, with significantly increased CRT. After two intravitreal anti-VEGF injections, BCVA increased to 75 letters. Fundus colour photo (**c**) and SD-OCT (**d**) showed that the fluid and scarring had subsided, with recovery of the foveal contour

**Fig. 2:**
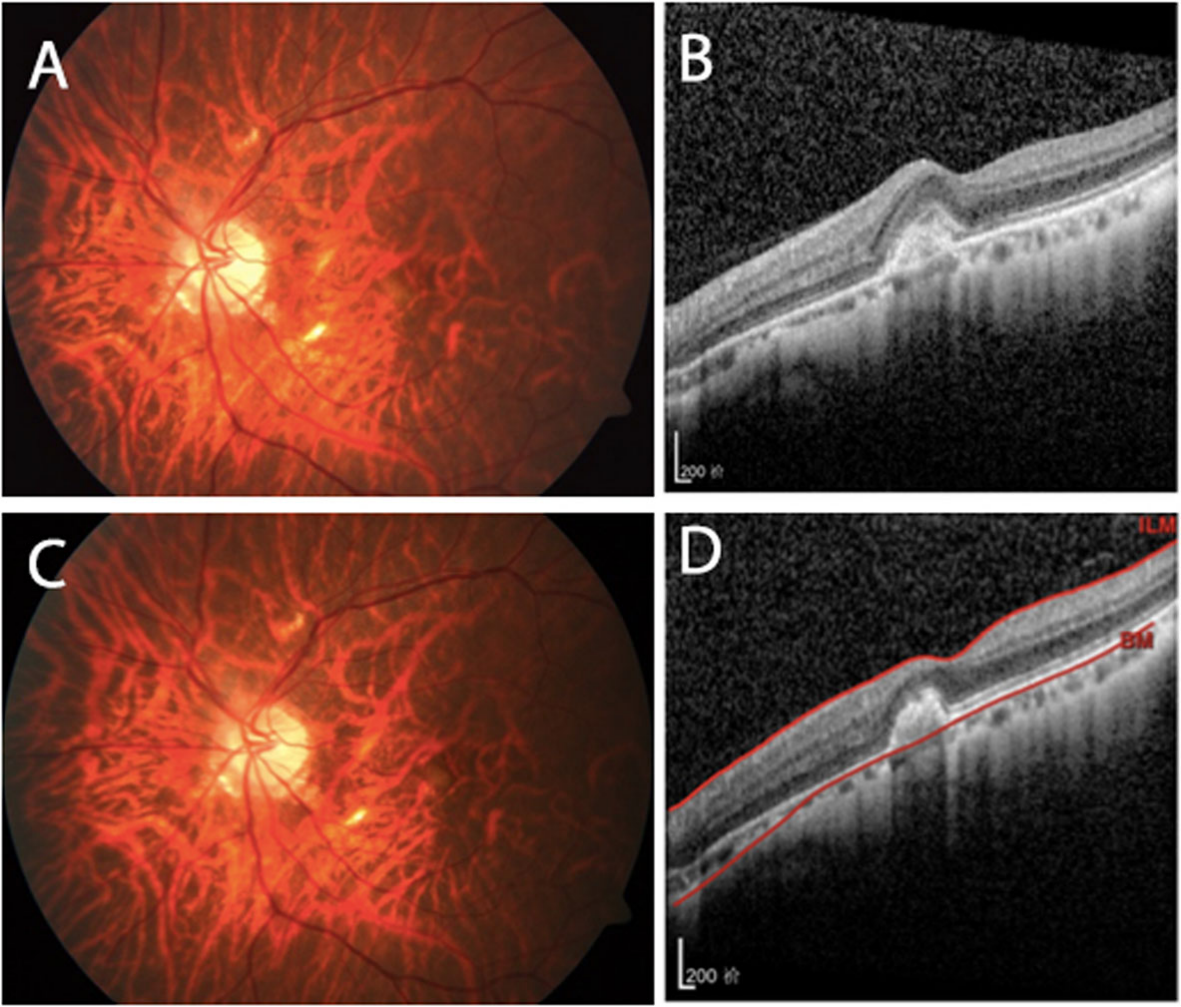
Left eye of a patient without morphological improvement after the first follow-up period. At baseline, BCVA was 53 letters and the colour fundus photo (**a**) showed a pigmentation disorder of the macular centre. SD-OCT (**b**) showed the CNV lesion at the centre of the macula, with subretinal fluid and CRT increased. After two intravitreal anti-VEGF injections, BCVA increased to 75 letters. The fundus colour photo (**c**) and SD-OCT (**d**) showed that the ICNV lesion remained

Patients were classified into two groups on the basis of the morphological improvement according to OCT reports at the end of the first follow-up period: patients with morphological improvement (Fig. 1) and patients without morphological improvement (Fig. 2). As shown in Table 3, there was no significant difference in age, gender, eyes, duration of disease, number of injections, and baseline CRT between the two groups (*P* > 0.05). Intravitreal bevacizumab and ranibizumab had similar effects on morphological changes, which indicated that the same effects on ICNV lesion subsided (*P* = 0.709). The baseline BCVA (63.00 ± 14.24) of patients with morphological improvement was significantly better than that of patients without morphological changes (52.18 ± 12.73; *P* = 0.026). After intravitreal anti-VEGF treatment, BCVA and CRT improvement were observed in both groups compared with baseline, but the differences were not statistically significant (*P* > 0.05).

**Table 3:** Comparison of parameters between patients with and without morphological improvement

Characteristics	Patients with morphological improvement (*n* = 13)	Patients without morphological improvement (*n* = 22)	*P*
Age(years)	34.46 ± 9.404	36.82 ± 9.620	0.544^a^
Gender(male/female)	7/6	7/15	0.288^b^
Eye(right/left)	3/10	10/12	0.282^b^
Anti-VEGF(bevacizumab/ranibizumab)	3/10	7/15	0.709^b^
Duration of disease(weeks)	21.53 ± 11.85	25.86 ± 18.04	0.775^c^
Number of injections(times)	2.23 ± 0.725	2.50 ± 0.802	0.335^c^
Baseline BCVA(letters)	63.00 ± 14.24	52.18 ± 12.73	0.026^a*^
Baseline CRT(μm)	347.9 ± 104.1	357.0 ± 97.73	0.649^c^
BCVA improvement(letters)	14.69 ± 13.51	18.55 ± 19.35	0.257^c^
CRT decrease(μm)	99.77 ± 89.55	69.18 ± 80.89	0.287^c^

^a^ Statistical analysis performed by the sample t-test

^b^ Statistical analysis performed by the Chi-square test

^c^ Statistical analysis performed by the Mann–Whitney U test

### Univariate logistic analysis of morphological improvement

The number of injections, baseline BCVA, age, baseline CRT, and duration of disease were included in a univariate analysis using morphological improvement as the dependent variable after the last injection in the first follow-up period. Factors affecting the efficacy of intravitreal anti-VEGF injections were analysed. Univariate analysis showed that the baseline BCVA had the strongest correlation with the morphological alterations after treatments (OR = 1.063, *P* = 0.036) (Table 4).

**Table 4:** Univariate analysis of morphological improvement

Characteristics	OR	95%CI	*P*
Number of injections(times)	0.629	0.253–1.566	0.319
Baseline BCVA(letters)	1.063	1.004–1.126	0.036^*^
Age(years)	0.973	0.904–1.048	0.473
Baseline CRT(μm)	0.999	0.992–1.006	0.788
Duration of disease(weeks)	0.980	0.931–1.032	0.444

### Comparison between subgroups according to baseline BCVA

Patients were divided into 2 groups on the basis of baseline BCVA: less than 60 letters (21 eyes) and better than 60 letters (14 eyes). The CRT of the patients whose baseline BCVA levels were less than 60 letters reduced by 66.71 μm at the end of the first follow-up period. The CRT of the patients whose baseline BCVA levels were better than 60 letters reduced by 101.3 μm at the end of the first follow-up period. Although there were no significant differences in CRT reduction between the two groups (*P* = 0.240), CRT was lower in the group with better baseline BCVA than in the group with poorer baseline BCVA (*P* = 0.013) after intravitreal anti-VEGF therapy (Table 5).

**Table 5:** Comparison of parameters between patients with different baseline BCVA levels

Characteristics	Baseline BCVA<60 letters	Baseline BCVA≥60 letters	*P*
Baseline CRT(μm)	355.8 ± 102.4	350.4 ± 96.50	0.877^a^
CRT change(μm)	66.71 ± 89.04	101.3 ± 74.79	0.240^a^
Final CRT(μm)	289.1 ± 56.04	249.1 ± 40.47	0.013^b*^

^a^ Statistical analysis performed by the sample t-test

^b^ Statistical analysis performed by the Mann–Whitney U test

### Recurrence

**Fig. 3:**
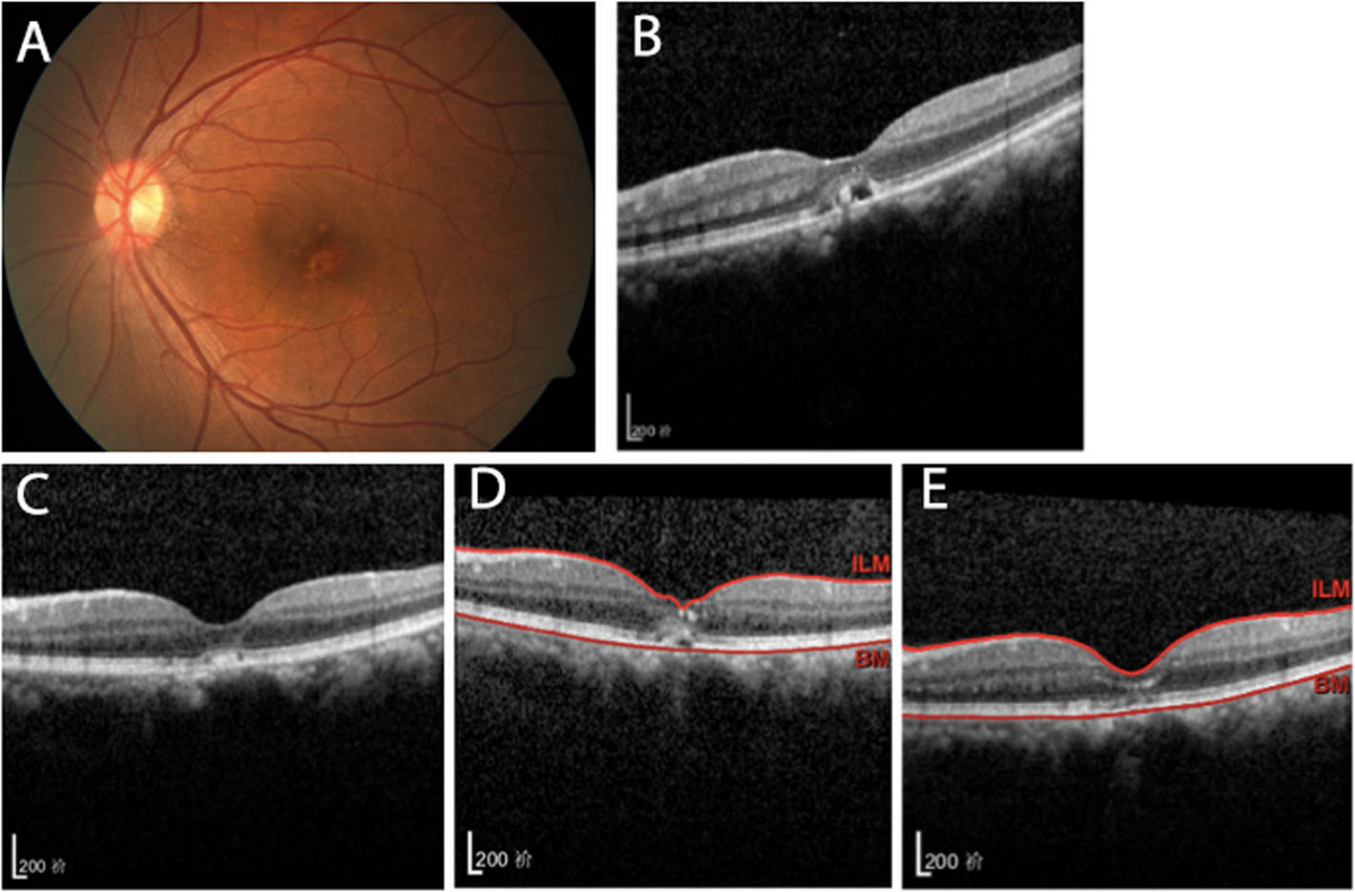
Left eye of a patient with morphological improvement after the first follow-up period and ICNV recurrence. At baseline, BCVA was 65 letters and the colour fundus photo (**a**) showed a pigmentation disorder of the macular centre. SD-OCT (**b**) showed CNV lesions with intraretinal fluid, and the structure of each layer of the retina was unclear. Five months later, after the second intravitreal injection, BCVA improved to 75 letters and OCT (**c**) showed that CNV lesions had subsided with recovery of foveal contour. Eighteen months after initial diagnosis, OCT (**d**) showed recurred intraretinal fluid, and the thickness of fovea increased significantly. One month after an additional intravitreal anti-VEGF therapy (**e**), the fovea returned to normal with the CNV lesions having completely subsided

**Fig. 4:**
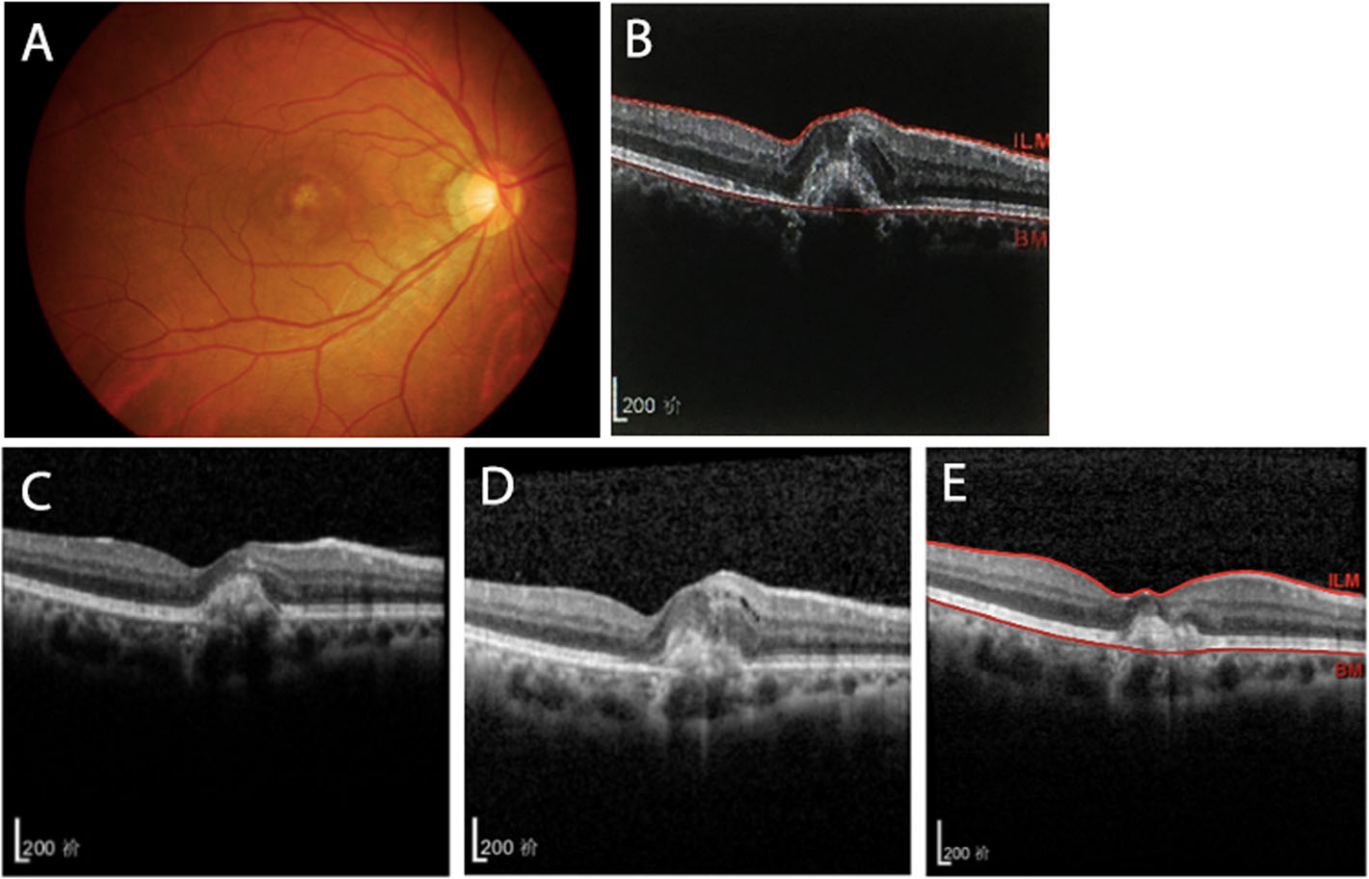
Right eye of a patient without morphological improvement after the first follow-up period and ICNV recurrence. At baseline, BCVA was 50 letters and the colour fundus photo (**a**) showed a pigmentation disorder of the macular centre. SD-OCT (**b**) showed CNV lesions with subretinal fluid, and the structure of each layer of the retina was unclear. Eleven months later, after the second intravitreal injection, BCVA improved to 69 letters and OCT (**c**) showed a scar. Fifteen months after initial diagnosis, BCVA decreased to 44 letters, and OCT (**d**) showed recurring subretinal fluid and a slightly increased thickness of the fovea. One month after an additional intravitreal anti-VEGF therapy (**e**), subretinal fluid had subsided and scarring remained

Patients were scheduled to visit the doctor every 12 weeks, and 8 patients failed to follow-up. The mean follow-up period was 168.0 ± 34.82 weeks (range: 111.5–259.7). The BCVA of the 27 patients at the first follow-up period (72.74 ± 13.97 letters, *P* < 0.001) and at final follow-up (73.59 ± 12.08 letters, *P* < 0.001) had significantly improved over the baseline (55.70 ± 15.21 letters). In this study, CNV recurred in 6 patients (Fig. 3, Fig. 4), 1 of whom experienced 2 recurrences. The recurrence rate was 22.22%. Recurrence was observed at a mean of 90.83 ± 49.20 weeks (range: 33–177) after diagnosis. There was no significant difference in age, type of anti-VEGF drugs, baseline BCVA, baseline CRT, final BCVA or final CRT between the two groups (Table 6, Table 7). There was no significant correlation between ICNV recurrence and the morphological changes at the end of the first follow-up period (χ^2^ = 0.622,*P*>0.05; Table 7).

**Table 6:** Comparison between patients with and without recurrence

Characteristics	Patients with recurrence (*n* = 6)	Patients without recurrence (*n* = 21)	*P*
Age (years)	37.50 ± 11.98	35.38 ± 8.755	0.634^a^
Baseline BCVA (letters)	49.67 ± 15.14	57.43 ± 15.15	0.279^a^
Baseline CRT (μm)	298.8 ± 63.87	349.6 ± 93.55	0.195^b^
Final BCVA (letters)	65.33 ± 16.68	75.95 ± 9.687	0.065^b^
Final CRT (μm)	246.2 ± 10.94	269.8 ± 52.63	0.195^b^
Duration of follow-up (weeks)	168.5 ± 53.43	167.9 ± 29.37	0.973^a^

^a^ Statistical analysis performed by the sample t-test

^b^ Statistical analysis performed by the Mann–Whitney U test

**Table 7:** Comparison of anti-VEGF therapy and morphological changes between patients with and without recurrence

	Eyes	Anti-VEGF therapy				Morphological changes			
		bevacizumab		ranibizumab		Patients with morphological improvement		Patients without morphological improvement	
		Eyes	n	Eyes	n	Eyes	n	Eyes	n
Patients with recurrence	6	3	50.0%	3	50.0%	1	16.7%	5	83.3%
Patients without recurrence	21	5	23.8%	16	76.2%	7	33.3%	14	66.6%
χ^2^		1.535				0.622			
*P*		0.319				0.633			

## Discussion

Comparative studies have shown promising outcomes of anti-VEGF therapy for ICNV, but few have demonstrated the long-term efficacy [7–10]. The aim of the present study was to investigate the prognostic factors and incidence of CNV recurrence related to retinal morphological changes of ICNV.

Previous studies have found that anti-VEGF therapy was effective in treating ICNV, recovery of visual acuity and CRT reduction [16, 17]. However, treatment regimen of intravitreal anti-VEGF therapy for ICNV was not well understood. Anti-VEGF therapy used at baseline and then PRN has been found comparable efficacy in most of the studies on ICNV [7, 9, 17, 18]. Previous studies [7, 15] have found that intravitreal bevacizumab on a PRN basis was effective in the treatment of ICNV and suggested that a single loading injection, followed by as-needed dosing, achieved favorable outcome. Taking Chinese current medical environment into account, we thought that the 1 + PRN regimen not only reduced the burden of patients’ costs, but also reduced the risk of systemic complications caused by repeated intravitreal injections [19]. In our study, the average number of injections was 2.40 ± 0.78 times, which was similar with the previous study in China [9]. At the end of the first follow-up period, BCVA increased by an average of 17 letters, and CRT decreased by 80 μm on average. Our study added to the evidence that 1 + PRN regimen achieved the favorable outcome in ICNV eyes. Sudhalkar A et al. [16] found no difference in the efficacy and safety between IVR and IVB in treating ICNV. Our study also found that there were no difference between IVR and IVB groups in BCVA and CRT at the end of the first follow-up period, which might indicate that IVB and IVR were equally effective in treating ICNV. However, the current study was retrospective and the sample size was relatively small. Our study stoped short of drawing that conclusion. Our results only excluded the influence of heterogeneous drugs in this study. Prospective study with a larger sample size would throw light on the efficacy of different anti-VEGF agents in ICNV.

Today, we often diagnose CNV, decide whether to perform the anti-VEGF treatment and monitor the effect of treatments according to OCT scans [20], because it is safe, quick and non-invasive [12]. Morphological improvement in OCT, which shows recovery of the foveal contour, indicates a good prognosis and better recovery of retinal function [21]. In this study, we used morphological changes in OCT as an important parameter. Our study indicated that a better baseline BCVA was associated with morphological improvement and we found that the better the baseline BCVA (more than 60 letters) was, the lower the CRT value after injections, and the more significant the resolution of foveal CNV. The results suggested that a timely treatment for ICNV when BCVA remained good might result in better resolution of ICNV lesions, recovery of the foveal contour, and better visual outcome. This was consistent with the study by Sudhalkar A et al. [16] which showed that patients with a higher baseline BCVA had a better prognosis. These findings might relate to the high permeability of neovascular structures, which could cause bleeding and exudation and induce inflammatory reactions and retinal damage [5, 22]. ICNV patients with a good baseline BCVA might not suffer from irreversible inflammatory effects and retinal damage; therefore, our results suggest that timely anti-VEGF treatment can effectively reduce the inflammatory response and make CNV lesions subside, in turn leading to a better prognosis.

Subsequently, we continued to observe the recurrence of the disease after the end of treatment. Kim JH et al. [23] reported a long-term follow-up of 26 patients with ICNV after intravitreal anti-VEGF treatment and the mean follow-up period was 33.9 months. The recurrence rate was 30.8%, and the recurrence of the disease was not related to age, baseline BCVA, or CRT. However the visual prognosis of patients with recurrence was not significantly different from that without recurrence. In our study, BCVA improved significantly at the end of the first follow-up period and lasted throughout the whole study period, suggesting a favorable outcome for anti-VEGF therapy used at baseline. The recurrence rate was 22.22%. The recurrent lesions had subsided after additional intravitreal anti-VEGF treatment. Compared with the non-recurrence group, the final BCVA of the recurrence group was not significantly different, suggesting that recurrent ICNV could be treated with additional anti-VEGF treatment and that recurrence had no significant effect on the final visual outcome, which was consistent with the results of Kim JH’s study [23]. At present, the indicators for diagnosing ICNV recurrence remained unclear. To the best of our knowledge, this study was the first that provided data on the relationship between ICNV recurrence and morphological outcomes of early treatment. We found that there was no correlation between the morphological changes and recurrence. In our study, patients with good morphological improvement after treatments also relapsed during long-term follow-up, suggesting that improvement in foveal morphology might not effectively predict the long-term prognosis. This may contribute to the formation of scars and decreased retinal function due to ICNV disease, which can lead to a poor visual prognosis [24]. Our study suggests that although patients with morphological improvement and recovery of the foveal contour seem to have a good prognosis, consequent monitoring should also be performed to avoid ICNV recurrence. However, our study just made recommendations because of the limited number of recurrence eyes and retrospective nature. Clinical trials with larger sample size are required to further evaluate the recurrence of ICNV.

There were limitations to this study. The study was retrospective and the follow-up schedule was not strict, resulting in the loss of some follow-up patients and heterogeneous follow-up period. Another limitation may be the relatively small sample size. Further clinical researches with larger sample sizes, controlled schedule and long-term follow-up period are necessary to explore predictors and outcomes for ICNV prognosis and recurrence.

## Conclusion

In conclusion, anti-VEGF therapy on a PRN basis achieved a favorable outcome in ICNV eyes. Patients with good baseline BCVA had a better prognosis. The morphological improvement after treatment had no significant correlation with recurrence, suggesting that subsequent monitoring should be performed in ICNV patients. Re-treatment with anti-VEGF could effectively lead to the resolution of recurrent ICNV and might not significantly influence the final visual outcome of the disease.

## Data Availability

The datasets used and analyzed during the current study are available from the corresponding author on reasonable request.

## Abbreviations

AMD: Age-related macular degeneration
BCVA: Best corrected visual acuity
CNV: Choroidal neovascularization
CRT: Central retinal thickness
CSC: Central serous chorioretinopathy
ICNV: Idiopathic choroidal neovascularization
IVB: Intravitreal bevacizumab
IVR: Intravitreal ranibizumab
OCT: Optical coherence tomography
PRN: Pro re nata
RPE: Retinal pigment epithelium
SD-OCT: Spectral-domain optical coherence tomography
VEGF: Vascular endothelial growth factor
